# The general anesthetic propofol induces ictal-like seizure activity in hippocampal mouse brain slices

**DOI:** 10.1186/s40064-015-1623-1

**Published:** 2015-12-24

**Authors:** Logan J. Voss, Liisa Andersson, Anna Jadelind

**Affiliations:** Anaesthesia Department, Waikato District Health Board, Pembroke St, Hamilton, 3240 New Zealand; School of Science and Engineering, University of Waikato, Knighton Road, Hamilton, 3240 New Zealand

**Keywords:** Ictal, General anesthesia, Propofol, Etomidate, Slice

## Abstract

The general anesthetic propofol has been in clinical use for more than 30 years and has become the agent of choice for rapid intravenous induction. While its hypnotic and anti-convulsant properties are well known, the propensity for propofol to promote seizure activity is less well characterised. Electroencephalogram-confirmed reports of propofol-induced seizure activity implicate a predisposition in epileptic subjects. The aim of this study was to investigate the seizure-promoting action of propofol in mouse brain slices—with the goal of establishing an in vitro model of propofol pro-convulsant action for future mechanistic studies. Coronal slices were exposed to either normal artificial cerebrospinal fluid (aCSF) or no-magnesium (no-Mg) aCSF—and extracellular field potential recordings made from the hippocampus, entorhinal cortex and neocortex. Propofol (and etomidate for comparison) were delivered at three stepwise concentrations corresponding to clinically relevant levels. The main finding was that propofol induced ictal-like seizures in seven out of ten hippocampal recordings (p = 0.004 compared to controls) following pre-exposure to no-Mg aCSF—but strongly inhibited seizure-like event (SLE) activity in the neocortex. Propofol did not induce seizure activity in slices exposed to normal aCSF. The results support the contention that propofol has the capacity to promote seizure activity, particularly when there is an underlying seizure predisposition. This study establishes an in vitro model for exploring the mechanisms by which propofol promotes subcortical seizure activity.

## Background

Propofol was introduced to clinical practice as a general anesthetic in 1977 and has become the agent of choice for rapid intravenous induction. It was originally thought to be a pure hypnotic (Samra et al. 1995), but has since been shown to have strong anticonvulsant properties (al-Hader et al. 1992)—the equal of thiopental for treating refractory status epilepticus (Prabhakar et al. 2012). The pro-convulsant potential of propofol has been debated and remains a controversial area (Samra et al. 1995).

Myoclonic activity during propofol anesthesia is common (Walder et al. 2002) and many case-studies report convulsive-like muscle activity, particularly during induction and emergence (Sutherland and Burt 1994). Electroencephalographic (EEG) confirmation of cortical seizure activity (Hodkinson et al. 1987; Makela et al. 1993; Wang et al. 1997) points to a dual action of propofol—predominantly anticonvulsant with a weaker pro-convulsant effect (Bevan 1993). Where the EEG has confirmed seizure activity, subjects with a history of epilepsy tend to be overrepresented. For example, Hodkinson et al. (1987) report three consecutive cases of propofol-induced epileptiform activity in the EEG of epileptic subjects. In a study reviewing five cases of convulsant-like muscle activity, the single EEG-confirmed case of seizure activity was from an epileptic (Makela et al. 1993). In a similar study, EEG spikes and sharp waves were recorded in epileptics and non-epileptics following low-dose propofol, but the one case of grand mal seizure activity was from the former (Wang et al. 1997). Accordingly, it has been recommended that propofol be avoided or used with caution in subjects with a history of epilepsy (Hodkinson et al. 1987; Makela et al. 1993; Wang et al. 1997).

Abnormal posturing and convulsive-like movements during propofol anesthesia are not universally associated with EEG changes consistent with cortical seizure activity (Borgeat et al. 1993). These could be instances of non-seizure-related muscle dystonia and/or epileptiform activity originating from deeper structures (Borgeat et al. 1993). It is noteworthy that subcortical seizure activity, which may present as myoclonic convulsive-like movements (Gale 1992), can be difficult to detect in the EEG (Borgeat et al. 1993; Kiloh and Osselton 1961; Makela et al. 1993). Furthermore, focal epileptiform events that are spatially limited may only be detectable with high-density electrocorticogram recordings. Thus, a normal EEG does not necessarily preclude an epileptiform aetiology.

With this as background, we designed a series of experiments to investigate the seizure-promoting action of propofol in mouse brain slices. The goal was to establish an in vitro model of propofol pro-convulsant action that could be probed for specific mechanisms in future studies. Three questions were of paramount interest. Firstly, could we identify a pro-seizure effect of propofol in the slice model? Secondly, could the apparent in vivo susceptibility of epileptics to propofol-induced seizures be replicated? Thirdly, are cortical or subcortical regions differentially sensitive to propofol seizure promoting effects? Slices were pre-exposed to no-magnesium conditions—initiating ongoing seizure-like events (SLE) akin to in vivo interictal activity—and compared to quiescent slices exposed to “normal” conditions. We focussed on three anatomical regions, the hippocampus, entorhinal cortex and the neocortex. The former is of particular interest as the site of seizure generation in temporal lobe epilepsy (Das et al. 2009) and as a documented target for anesthetic excitatory effects (Becker et al. 2012). The entorhinal area is also a seizure-prone region by nature of its recurrent network structure (Dhillon and Jones 2000). Finally, we compared the effect of propofol to that of etomidate, which shares with propofol a gamma-aminobutyric acid (GABA)-receptor mechanism of action (Lingamaneni and Hemmings 2003). We choose etomidate as a comparator because it is recognised as having pro-seizure characteristics (Modica et al. 1990) and because it is the most specific GABAergic anesthetic (Franks 2006).

## Results and discussion

### Baseline SLE characteristics

**Fig. 1:**
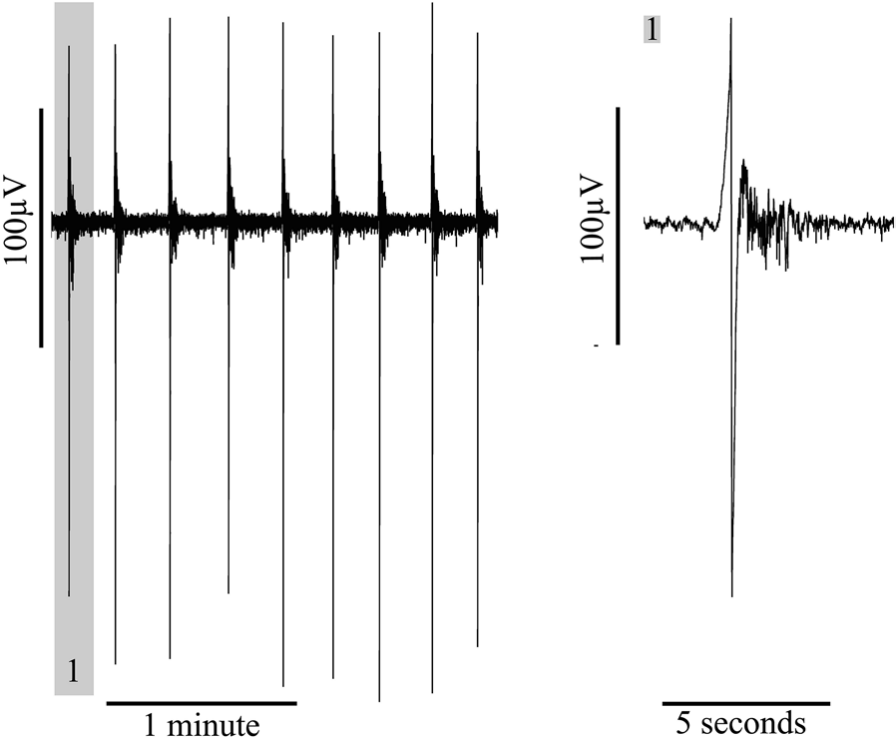
An example of no-magnesium seizure-like event (SLE) activity showing an expanded time view on the *left* and an enlarged view of a single SLE on the *right*

Slices perfused with no-Mg aCSF generated spontaneous interictal-like SLE activity characterised by a sharp positive or negative deflection in the field potential of approximately 100 μV peak–peak amplitude and of 1–2 s duration (illustrated in Fig. 1). On some occasions this initial population “spike” was followed by oscillatory activity of lower amplitude, lasting up to approximately 5 s in the neocortex (see Tables 1, 2). Longer oscillatory activity was recorded in the entorhinal cortex compared to both the hippocampus (p < 0.001) and the neocortex (p < 0.05). The frequency of baseline SLE activity was significantly higher in recordings from the hippocampus compared to the entorhinal cortex (p < 0.001) and the neocortex (p < 0.001). No statistical difference was seen in baseline amplitude between the different brain regions.

**Table 1:** Median (range) changes in SLE-characteristics during propofol administration

Location	Baseline	Last dose	End period	P value
Entorhinal cortex				
Amplitude (µV*)*	92.5 (23.7–341.9)	111.3 (25.9–310)	96 (36.6–429)	ns
Frequency (/min)	2.0 (1.3–4.9)	0.7 (0.3–1.1)^a^	0.7 (0.3–1.4)^a^	<0.0001*
Length (s)	3.6 (1.1–9.9)	9.1 (1.0–51.5)	18.9 (1.1–49)^b^	0.0155*
Neocortex				
Amplitude (µV)	138.7 (37–494)	245.7 (34.3–615)^c^	229.4 (38.8–429.2)	0.0302*
Frequency (/min)	2.5 (0.6–4.4)	0.6 (0.0–3.6)^d^	0.7 (0.1–4.5)^d^	<0.0001**
Length (s)	2.3 (1–5.1)	1.9 (0–12.6)	1.8 (1–6.5)	ns

* p values generated from repeated measures analysis of variance (ANOVA)

** p values generated from Freidman test (nonparametric repeated measures ANOVA)

^a^p < 0.001 compared to baseline, Tukey–Kramer multiple comparison test

^b^p < 0.05 compared to baseline, Tukey–Kramer multiple comparisons test

^c^p < 0.05 compared to baseline, Dunn’s multiple comparisons test

^d^p < 0.001 compared to baseline, Dunn’s multiple comparisons test

**Table 2:** Median (range) changes in SLE-characteristics during etomidate administration

Location	Baseline	Last dose	End period	P value
Entorhinal cortex				
Amplitude (µV*)*	79.9 (28.3–229.8)	95.7 (20.8–172.9)	108.0 (30.0–199.3)	ns
Frequency (/min)	2.7 (0.4–4.0)	1.2 (0.2–1.9)^a^	2.7 (0.5–6.5)^b^	0.0004*
Length (s)	2.9 (2.1–143.3)	2.5 (1.0–246.6)	3.4 (1.3–73.7)	ns
Neocortex				
Amplitude (µV*)*	136.6 (46.8–502.6)	178.5 (75.0–663)^c^	141.2 (67.4–633.0)	0.0084**
Frequency (/min)	3.4 (3.2–5.2)	1.3 (0.3–3.4)	3.7 (0.7–12.7)	0.0515**
Length (s)	2.2 (1.2–3.4)	1.5 (1.1–1.7)	1.7 (1.5–3.6)	ns
Hippocampus				
Amplitude (µV*)*	90.1 (11.8–280.5)	103.8 (11.9–428.6)	135.1 (14.3–346.6)^d^	0.0398*
Frequency (/min)	5.8 (3.5–7.9)	2.1 (0.1–5.1)^c^	4.5 (2.3–17.6)	0.0013**
Length (s)	1.1 (1.0–3.2)	1.2 (0.9–1.7)	1.2 (1.0–3.2)	ns

* p values generated from repeated measures analysis of variance (ANOVA)

** p values generated from Freidman test (nonparametric repeated measures ANOVA)

^a^p < 0.01 compared to baseline, Tukey–Kramer multiple comparisons test

^b^p < 0.001 compared to last dose, Tukey–Kramer multiple comparisons test

^c^p < 0.01 compared to baseline, Dunn’s multiple comparisons test

^d^p < 0.05 compared to baseline, Dunn’s multiple comparisons test

### Effects of propofol on no-Mg SLE activity

**Fig. 2:**
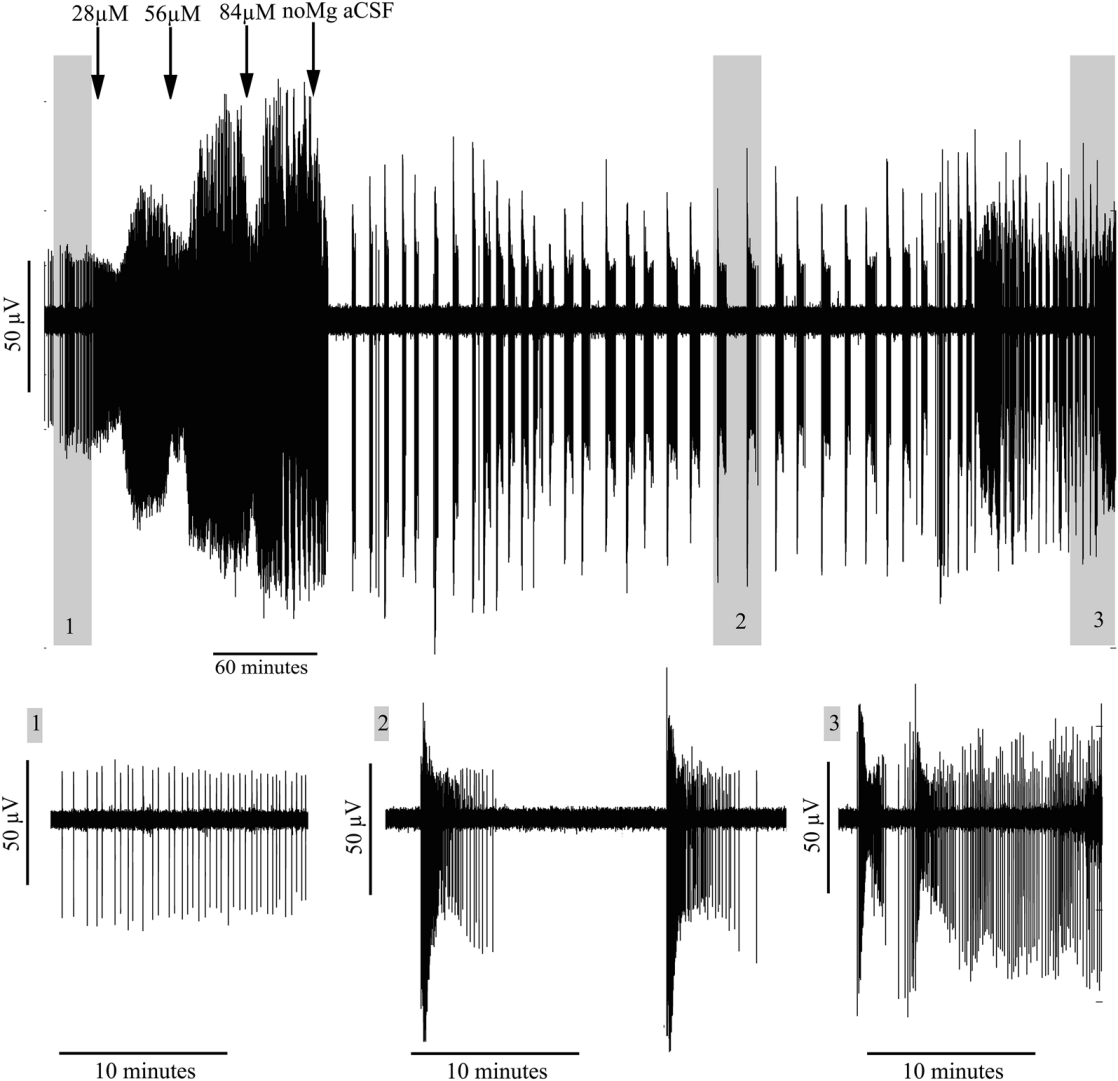
Propofol-induced ictal-like bursting activity in the hippocampus showing a changing pattern of the frequency and amplitude of population activity within each burst. The *top illustration* shows a full recording of approximately 9 h. The *three* *lower pictures* are enlargements of the *grey squares* and illustrate: *1* interictal seizure-like event (SLE) activity before propofol administration; *2* ictal-like burst during propofol administration with a biphasic change in frequency and amplitude and; *3* end period where the SLE-activity is returning to the interictal SLE-activty pattern established during baseline

Recordings during propofol administration showed highly variant changes in activity in the three investigated areas. Most dramatically, in the hippocampus propofol induced a transition from interictal-like SLE activity characterised by intermittent unitary sharp waves—to prolonged ictal-like seizure events. The characteristics of the latter were not identical in all hippocampal recordings, but two different groups with similar patterns could be identified. In one group (n = 4) the events started with a period of high frequency “tonic-like” activity that transitioned into “clonic-like” oscillations before terminating (see Fig. 2). On average one event occurred every 2–3 min, with a mean length of 196 s. In the other group (n = 3), oscillation frequency tended to remain stable throughout the event. They also occurred every 2–3 min, but had a shorter mean length of 37 s. Following drug washout, the majority of the recordings returned to the interictal-like activity pattern that was established during baseline. Seven out of ten of the propofol hippocampal recordings exhibited this transition to ictal-like seizure activity, compared with none of eight controls perfused with drug-free no-Mg aCSF (p = 0.004, Fisher’s exact test). No ictal-like events occurred in slices perfused with propofol vehicle (20 % intralipid).

**Fig. 3:**
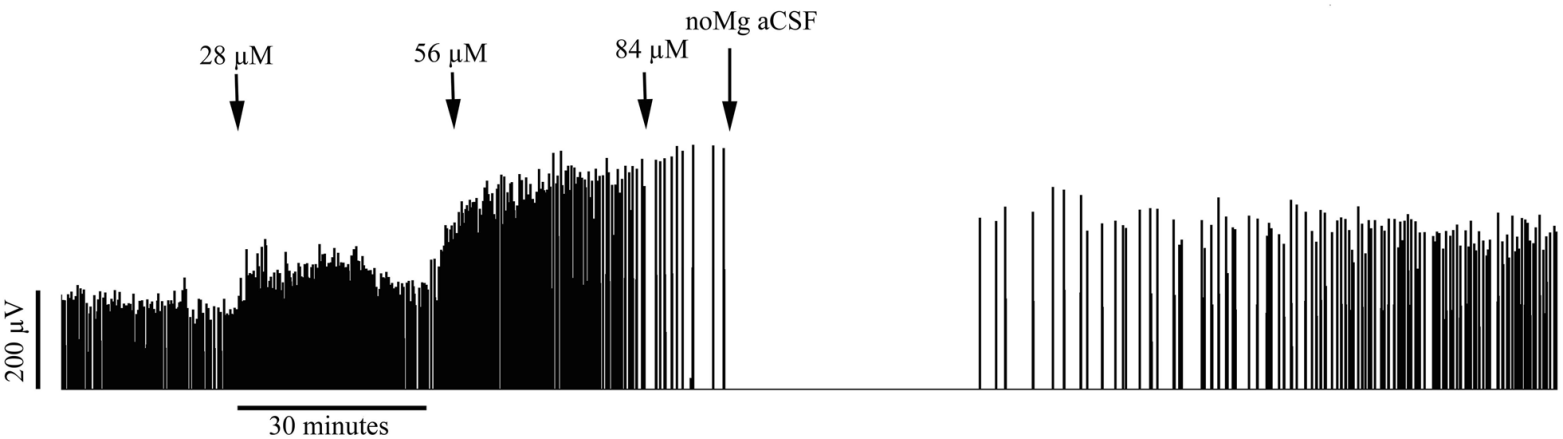
A full length recording showing the inhibitory effect of propofol on interictal seizure-like event (SLE) activity in the neocortex. This recording further illustrates the increase in event amplitude prior to cassation of activity

In the two other investigated areas, the entorhinal cortex and the neocortex, no transition to ictal-like seizures were seen during propofol perfusion. A strong decrease in event frequency was seen after drug administration in both the entorhinal cortex (p < 0.0001) and the neocortex (p < 0.0001) (Table 1). Figure 3 illustrates the profound inhibitory effect of propofol on cortical interictal SLE activity; compared with the ictal events precipitated in the hippocampus (see Fig. 2). Propofol had contrasting effects on SLE length and amplitude in the entorhinal and neocortical regions. In the former, propofol increased the length of SLEs (p = 0.0155), while in the neocortex it induced a significant (p = 0.0302) increase in SLE amplitude. These changes were not seen in the control slices perfused with no-Mg aCSF without drug delivery. Rather, SLE frequency during control recordings tended to increase with time, in keeping with previous studies (Voss et al. 2010).

### Effects of etomidate on no-Mg SLE-activity

One out of ten hippocampal recordings showed a transition to an ictal-like seizure pattern after etomidate administration, a rate not significantly different from the control recordings. The ictal-like pattern had the same characteristics as shown in Fig. 2 for propofol.

Otherwise, etomidate had a similar effect on no-Mg SLE activity in all three brain areas (see Table 2). A reduction in event frequency was seen in all three locations, although for the neocortex the frequency drop was not quite significant (p = 0.0515), due to a paradoxical increase in SLE frequency in one recording during perfusion of the highest etomidate dose. Like propofol, etomidate induced a significant increase in SLE amplitude in the neocortex (p = 0.0084), as well as in the hippocampus (p = 0.0398). Etomidate induced no significant changes in the length of SLEs in any location. None of these changes were seen in any of the control slices perfused with no-Mg without drug delivery.

### Propofol and etomidate effects on slices perfused with normal aCSF

Neither propofol nor etomidate induced any sign of SLE activity in recordings from slices perfused with normal aCSF. That is, these slices remained quiescent for the full duration of the recordings, at all locations.

The aim of this study was to investigate whether pro-seizure effects of the general anesthetic propofol could be identified in the in vitro cortical slice—and whether seizure susceptibility was related to the prior state of the tissue and/or anatomical location. Most notably, propofol consistently induced prolonged ictal-like seizure events in the hippocampus following pre-exposure to no-Mg aCSF. The hippocampal ictal-like events closely resembled tonic–clonic seizures recorded from the electroencephalogram in vivo (Jensen and Yaari 1997). The results support in vivo findings showing that propofol has the capacity to promote seizure activity—and suggest that subcortical regions may be particularly susceptible. The pro-seizure effects were observed on a background of epileptiform “spiking” activity induced by magnesium removal from the aCSF, a state that can be likened to the interictal epileptic brain. Thus, the results are also consistent with in vivo studies showing that propofol is more likely to promote epileptiform activity when there is an underlying seizure predisposition (Hodkinson et al. 1987; Makela et al. 1993; Wang et al. 1997). In addition, propofol strongly inhibited SLE activity in the neocortex, confirming its dual anti- and pro-convulsant properties (Bevan 1993).

By comparison, etomidate evoked few excitatory effects within the slice. It shared with propofol a tendency to enhance SLE amplitude in the neocortex and hippocampus, but otherwise reduced the frequency of SLE activity in all investigated areas. This was surprising on two levels. Firstly, etomidate and propofol are thought to share a similar GABAergic mechanism of action (Franks 2006); and secondly, etomidate is more well-known than propofol for its pro-seizure effects (Modica et al. 1990). An explanation for this difference is not forthcoming from this study, although a number of speculative hypotheses could be postulated. These include, but are not limited to: localised hippocampal expression of GABA_A_ subtypes that are differentially sensitive to etomidate and propofol; differences in the capacity for propofol and etomidate to directly activate GABA_A_ receptors and; differential effects of propofol and etomidate on NaKCC1 and KCC2 chloride transporters such that a chloride imbalance is facilitated by the former. However, the balance of evidence does not lend strong support to any one of these. For example, while α4 and α5-containing GABA_A_ receptors show enhanced expression in the hippocampus compared to the cerebral cortex (Sieghart and Sperk 2002), etomidate and propofol exhibit similar activation profiles for these GABA_A_ subtypes (Bai et al. 2001; Caraiscos et al. 2004; Meera et al. 2009). Furthermore, both propofol and etomidate directly activate GABA_A_ to approximately 25 % of the maximum current induced by endogenous GABA (Siegwart et al. 2002). Finally, the only documented effect of propofol on chloride handling is facilitation of KCC2 (Wang et al. 2006), which would tend to limit the development of depolarising GABA_A_ effects.

Although widely thought to act specifically on GABA_A_ receptors, propofol has additional actions that could contribute to its propensity to induce excitatory effects. Three possibilities present themselves. Firstly, reports indicate that glutamatergic effects may be relevant to propofol’s mechanism of action (Yiqing et al. 2014). In particular, propofol increases GluA1 AMPA receptor phosphorylation at serine 831 (S831) in the mouse hippocampus but not in the prefrontal cortex (Mao et al. 2014). S831 phosphorylation in GluA1 AMPA-receptors increases single channel conductance (Jenkins and Traynelis 2012) and potentiates LTP (Wang et al. 2014). This represents a promising, but as yet untested potential mechanism for propofol-mediated, hippocampal-specific excitatory effects. Secondly, Schwieler et al. have shown that propofol is an agonist of metabotropic GABA_B_-receptors (Schwieler et al. 2003). Interestingly, baclofen (a GABA_B_ agonist) inhibits hippocampal inter-ictal activity, but in so doing facilitates ictal-like activity (Swartzwelder et al. 1987), an effect remarkably similar to that observed in our study. We have shown in unrelated experiments that the GABA_B_ agonist baclofen enhances the length of SLE activity in the neocortex (unpublished data). An excitatory GABA_B_ effect could be mediated by a pre-synaptic GABA_B_ autoreceptor action that acts to inhibit the release of GABA (Dugladze et al. 2013). Finally, propofol inhibits hyperpolarisation-activated cation currents (I_h_) in both cortical (Chen et al. 2005) and hippocampal (Funahashi et al. 2004) regions. This is an important point of distinction between propofol and etomidate, as the latter does not block I_h_ (Chen et al. 2009). A reduction in I_h_ has been implicated as a mechanism supporting epileptogenesis (Wierschke et al. 2010), which could occur secondary to an increase in neuronal input resistance resulting in enhanced EPSP amplitude (Magee 1998); and/or a loss of membrane stabilisation resulting in prolongation of oscillatory field potential activity (He et al. 2014).

The no-magnesium in vitro slice model was used in this study because it is a recognised and widely used method to investigate mechanisms of epilepsy (Isaev et al. 2012; Liu et al. 2010). In particular, the pattern of no-magnesium SLE activity resembles the interictal state in epileptics. The inherent limitations of an in vivo animal model such as this are countered by the ability to stringently control experimental conditions and isolate effects and mechanisms to particular structures. In the case of this study it allowed us to differentiate effects to the hippocampus and cerebral cortex in complete isolation from subcortical influences.

## Conclusions

In conclusion, the results support the contention that propofol has the capacity to promote seizure activity, particularly in the hippocampus and when there is an underlying seizure predisposition. The mechanism by which this occurs is not clear, but appears to be independent of the seizure-promoting action of etomidate. This study establishes a model for exploring the mechanisms by which propofol promotes subcortical seizure activity.

## Methods

The method of euthanasia was approved by the Waikato Ethics Committee at the University of Waikato, Hamilton, New Zealand.

### Preparation of slices

Brain slices were investigated from 30 wild type mice of C57, 129SV and C57/129SV genetic background. The animals were 1–6 months old, both sexes and kept with unlimited access to water and food in a 12-h day/night cycle. All age ranges were represented in all experimental groups.

The mice were anesthetised with carbon dioxide and the brain dissected and transferred directly to ice-cold carbogenated (95 % O_2_, 5 % CO_2_) “normal” artificial cerebrospinal fluid (aCSF) composed of 125 mM NaCl, 2.5 mM KCl, 1 mM MgCl_2_, 2 mM CaCl_2_, 1.25 mM NaH_2_PO_4_, 26 mM NaHCO_3_ and 10 mM glucose. To maximise the number of slices containing the hippocampus, the brain was sectioned (400 μm, Campden Instruments Ltd, Sileby, Leics, UK) coronally between Bregma 0 to −5 mm. Thereafter, the slices were allowed at least 1 h of recovery at room temperature (19–23 °C) in either normal aCSF or magnesium-free (no-Mg) aCSF, depending on the experiments. No–Mg aCSF was composed of 124 mM NaCl, 5 mM KCl, 2 mM CaCl_2_, 1.25 mM NaH2PO4, 26 mM NaHCO3 and 10 mM glucose. The aCSF solutions were used for no longer than 1 week following storage at 1–4 °C.

### Electrical recording of field potential activity

**Fig. 4:**
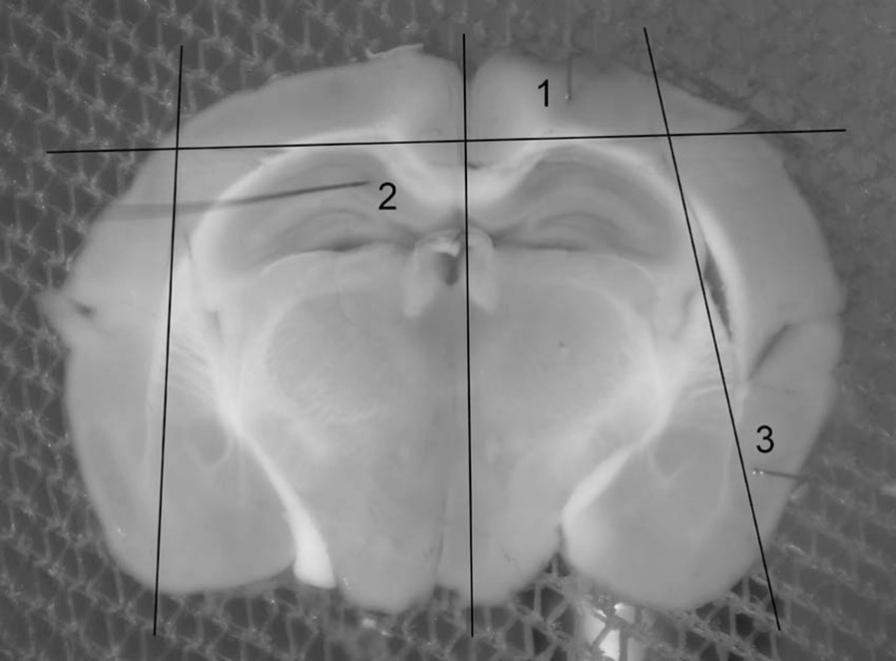
Picture of a mouse brain slice with each of the recording regions numbered adjacent to the respective electrodes. The *lines* illustrate where the slice was physically sectioned to isolate the cortex from the hippocampus. *1* the neocortex, *2* the hippocampus and *3* the entorhinal cortex

After recovery, one slice at a time was physically divided using a scalpel blade to isolate the cerebral cortex from the hippocampus; and one hemisphere from the other (see Fig. 4). This was to ensure that recordings from the cerebral cortex were independent of the hippocampus and vice versa. Each slice was then transferred to a submersion-style bath perfused with carbogenated normal or no-Mg aCSF, depending on the experiment being performed. Perfusion was by gravity feed at a flow rate of 5 ml/min. The flow rate was checked repeatedly during the recordings to ensure it remained stable.

Extracellular field potential activity was recorded from one to three 25 µm Teflon-coated tungsten electrodes, positioned in the neocortex, entorhinal cortex and hippocampus. For the neocortex no particular subregion was targeted and included the posterior parietal association, retrosplenial, primary somatosensory, primary motor, piriform, visual and auditory cortical areas. In the hippocampus electrodes were placed in the CA1, CA3 or dentate gyrus areas.

Recordings were amplified (1000×, A-M Systems, USA or 250× Kerr Tissue Recording System, Kerr Scientific Instruments, Christchurch, New Zealand), AD-converted (Power 1401, CED, UK or PowerLab, ADInstruments, Sydney, Australia) and stored on computer (Spike2, CED, UK or LabChart, ADInstruments, Sydney, Australia) for later offline analysis using MatLab software. The recordings were low-pass filtered at either 1000 or 100 Hz (CED and PowerLab, respectively) and high-pass filtered at 1 Hz. All of the experiments were performed in a Faraday shielded room to minimize electrical noise in the recordings.

### Drug preparation and delivery

Propofol was prepared as a 1 % solution of 2,6 diisopropylphenol 97+ % (SAFC supply solutions, USA) in 20 % Intralipid (Fresenius Kabi AB, Sweden) and etomidate as the commercially available Hypnomidate solution (Janssen-Cilag, Belgium). The drug concentrations used were 28, 56 and 84 μM for propofol and 8, 16 and 24 μM for etomidate. In each case the appropriate amount of each drug was added directly to pre-carbogenated aCSF. The concentrations were calculated to give tissue levels similar to that achieved clinically for surgical anesthesia, based on the diffusion charactersitics of etomidate into brain slice tissue and the relative clinical potencies of etomidate and propofol. Thus, at a slice recording depth of 100–200 μm, it takes approximately 10–15 min for the tissue concentration of etomidate to reach half of the drug concentration in the perfusion bath (Benkwitz et al. 2007). For our lowest etomidate concentration this equates to a tissue concentration of 4 μM. In rodents, a brain effect-site etomidate concentration of 13 μM induces a surgical level of anesthesia (De Paepe et al. 1999). Our relatively higher propofol concentrations were based on clinical data showing that etomidate is approximately five times more potent than propofol (Avramov et al. 1995).

### Experimental protocols

**Fig. 5:**
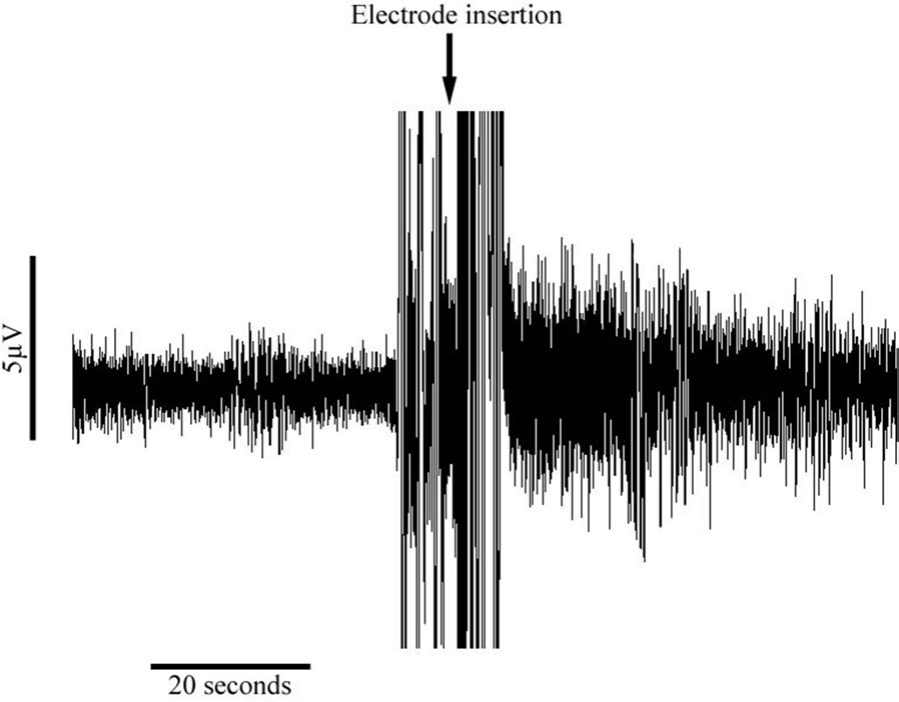
Illustration from one slice perfused with normal artificial cerebrospinal fluid (aCSF) showing a burst of high frequency activity upon electrode insertion. This characteristic response was used as a gauge of tissue viability in slices perfused with normal aCSF, which do not ordinarily express seizure-like event activity

Experiments were performed in either normal or no-Mg aCSF. In the case of the former, no baseline SLE activity was established in the tissue, according to standard practice. Under these conditions, viable tissue shows a transient burst of high frequency activity (see Fig. 5) when an electrode is inserted into the tissue (Voss et al. 2013). All recordings in normal aCSF showed this characteristic response. When using no-Mg aCSF, SLE activity was already established in the tissue and recordings were made from locations where robust and stable activity was found. In these cases, at least 10 min of stable SLE activity was recorded prior to delivery of the first anesthetic concentration. A corresponding “baseline” time-period was allowed for slices in normal aCSF. Thereafter, either propofol (n = 27 in no-Mg and n = 7 in normal aCSF) or etomidate (n = 19 in no-Mg and n = 12 in normal aCSF) were delivered at three increasing concentrations at 30 min intervals.

The no-Mg experiments were terminated when either the maximum anesthetic concentration was reached or SLE frequency dropped to 50 % of that established during baseline. When one of these criteria was met for all recordings in the slice, wash-out with drug-free aCSF followed. Wash out was continued for 40 min or until the return of SLE activity could be confirmed.

To control for the possibility of time effects: 3 slices were perfused with no-Mg aCSF with addition of three consecutive doses of drug-free 20 % intralipid solution equivalent in amount to the propofol experiments; and 13 slices were perfused with no-Mg aCSF for at least 3 h without delivery of anesthetic.

All slices were photographed and recording locations identified in consultation with the Allen Interactive Brain Atlas.

### Statistical analysis

For 36 out of a total of 178 recording locations, the protocol was not completed for one or other of the following reasons:

1. 10 min of stable baseline no-Mg SLE activity was not achieved.
2. Return of stable no-Mg SLE activity was not achieved during drug wash out,
3. A burst of activity with electrode insertion could not be identified in normal aCSF recordings.
4. Stable no-Mg SLE activity could not be achieved for a minimum of three hours during control recordings.
5. Electrodes were inadvertently moved during recording.

These recordings were excluded from the study, giving a total of 142 recordings for statistical analysis.

During the course of the experiments, it became apparent that propofol was inducing a unique pattern of activity in the majority of hippocampal recordings, characterised by a transition from interictal-like SLE activity into long ictal-like bursts (see “Results and discussion” for details). The propofol hippocampal data was therefore analysed as a separate group by quantifying the proportion of recordings exhibiting ictal-like bursting versus interictal-like SLE activity using the Fisher’s exact test. The qualitative characteristics of these recordings were assessed in detail.

The remainder of the data were analysed in MatLab for changes in SLE amplitude, frequency and length. Background noise and artefacts were first removed by visual inspection. The data were then quantified and averaged over three time periods covering:

1. *Baseline* 10 min before drug delivery.
2. *Last dose* 30 min during perfusion of either the maximum drug concentration or that concentration required to effect a 50 % reduction in SLE frequency. The 30 min analysis period was offset by 10 min from the start of perfusion of the relevant concentration, since at a flow rate of 5 ml/min, 10 min is needed for the drug concentration in the perfusion bath to equilibrate (data not shown).
3. *End period* the last 30–40 min of recording following drug washout.

In the control slices, corresponding time frames were selected. For a small proportion of recordings (10 out of 142) the automated MatLab scripts failed to accurately report SLE length. In these cases SLE length was manually quantified and averaged across five sequential SLEs corresponding to equivalent time points in the recordings.

Graphpad (GraphPad Software Inc 2003, v3.06) was used for all statistical analyses. Data normality was tested using the Kolmogorov–Smirnov test, and parametric or non-parametric ANOVA and pair-wise tests applied as appropriate. The data are presented as median (range). A *P* value <0.05 was considered statistically significant.
